# Diagnostic reliability of the Berlin classification for complex MCA aneurysms—usability in a series of only giant aneurysms

**DOI:** 10.1007/s00701-020-04565-6

**Published:** 2020-09-15

**Authors:** Lars Wessels, Lucius Samo Fekonja, Johannes Achberger, Julius Dengler, Marcus Czabanka, Nils Hecht, Ulf Schneider, Dimitri Tkatschenko, Karl-Michael Schebesch, Nils Ole Schmidt, Dorothee Mielke, Henning Hosch, Oliver Ganslandt, Alexander Gräwe, Bujung Hong, Jan Walter, Erdem Güresir, Philippe Bijlenga, Julien Haemmerli, Nicolai Maldaner, Serge Marbacher, Ville Nurminen, Hynek Zitek, Ruben Dammers, Naoki Kato, Italo Linfante, Maria-Teresa Pedro, Karsten Wrede, Wei-Te Wang, Maria Wostrack, Peter Vajkoczy

**Affiliations:** 1 Department of Neurosurgery and Center for Stroke Research Berlin (CSB), Charité Universitätsmedizin Berlin, Charitéplatz 1, 10117 Berlin, Germany; 2 Department of Neurosurgery, Helios Clinic, Bad Saarow, Germany; 3 Brandenburg Medical School Theodor Fontane, Campus Bad Saarow, Germany; 4 Department of Neurosurgery, University of Regensburg, Regensburg, Germany; 5 Department of Neurosurgery, University Medical Center Hamburg-Eppendorf, Hamburg, Germany; 6 Department of Neurosurgery, Georg-August-University Goettingen, Göttingen, Germany; 7 Department of Neurosurgery, Vivantes Klinikum im Friedrichshain, Berlin, Germany; 8 Department of Neurosurgery, Klinikum Stuttgart, Germany; 9 Department of Neurosurgery, Unfallkrankenhaus Berlin, Germany; 10 Department of Neurosurgery, Hannover Medical School, Hannover, Germany; 11 Department of Neurosurgery, University Hospital Jena, Jena, Germany; 12 Department of Neurosurgery, Medical Center Saarbrücken, Saarbrücken, Germany; 13 Department of Neurosurgery, University Hospital Bonn, Bonn, Germany; 14 Service de Neurochirurgie, Faculté de Médecine de Genève and Hôpitaux Universitaire de Genève, Geneva, Switzerland; 15 Department of Neurosurgery, University Hospital of Zurich, Zürich, Switzerland; 16 Department of Neurosurgery, Kantonsspital Aarau, Aarau, Switzerland; 17 Neurosurgery, University of Helsinki and Helsinki University Hospital, Helsinki, Finland; 18 Department of Neurosurgery, J. E. Purkinje University, Masaryk Hospital, Ústí nad Labem, Czech Republic; 19 Erasmus Stroke Center, Erasmus MC University Hospital, Rotterdam, The Netherlands; 20 Department of Neurosurgery, Jikei University School of Medicine, Tokyo, Japan; 21 Interventional Neuroradiology and Endovascular Neurosurgery at Miami Cardiac and Vascular Institute and Baptist Neuroscience Institute, Miami, USA; 22 Department of Neurosurgery, University Hospital of Ulm, Ulm, Germany; 23 Department of Neurosurgery, University of Essen, Duisburg, Germany; 24 Department of Neurosurgery, Medical University, Vienna, Austria; 25 Department of Neurosurgery, Technical University of Munich, Munich, Germany

**Keywords:** Giant aneurysm, Cerebral bypass, MCA aneurysm

## Abstract

**Background and objective:**

The main challenge of bypass surgery of complex MCA aneurysms is not the selection of the bypass type but the initial decision-making of how to exclude the affected vessel segment from circulation. To this end, we have previously proposed a classification for complex MCA aneurysms based on the preoperative angiography. The current study aimed to validate this new classification and assess its diagnostic reliability using the giant aneurysm registry as an independent data set.

**Methods:**

We reviewed the pretreatment neuroimaging of 51 patients with giant (> 2.5 cm) MCA aneurysms from 18 centers, prospectively entered into the international giant aneurysm registry. We classified the aneurysms according to our previously proposed Berlin classification for complex MCA aneurysms. To test for interrater diagnostic reliability, the data set was reviewed by four independent observers.

**Results:**

We were able to classify all 51 aneurysms according to the Berlin classification for complex MCA aneurysms. Eight percent of the aneurysm were classified as type 1a, 14% as type 1b, 14% as type 2a, 24% as type 2b, 33% as type 2c, and 8% as type 3. The interrater reliability was moderate with Fleiss’s Kappa of 0.419.

**Conclusion:**

The recently published Berlin classification for complex MCA aneurysms showed diagnostic reliability, independent of the observer when applied to the MCA aneurysms of the international giant aneurysm registry.

## Introduction

Besides improvements in endovascular techniques and versatile clipping strategies, there are still some complex aneurysms—giant, fusiform, or partially thrombosed/calcified—with a need for vessel sacrifice after revascularization of the downstream vessel segment [10, 13, 14, 19, 23]. Complex aneurysms of the middle cerebral artery (MCA) are a special challenge. This is due to multiple perforators in the M1 segment and the difficult accessibility of M1 and M2 branches, especially in very large aneurysms [6].

For the preoperative planning of cerebral revascularization and aneurysm occlusion, it is of importance to anticipate the intraoperative anatomy. Besides new developments in the field of 3D reconstruction CT angiography, digital subtraction angiography remains the best solution to estimate the orientation of the aneurysm to the vessel branches [1, 8].

The preoperative planning has to address two questions. The first question aims at determining the strategy of how to handle the aneurysm. Ideally, the goal should be to trap the aneurysm, but sometimes, this is not possible without placing the patient at risk of perforator ischemia or due to inaccessibility of the inflow or outflow segments. In cases where trapping is not an option, fallback strategies are proximal or distal occlusion. The second question to answer is what kind of revascularization is necessary, depending on the strategy chosen for aneurysm occlusion.

The pertinent literature tries to propose a clear recommendation for the technique to use for revascularization and aneurysm occlusion [20]. But since there is a large armamentarium for surgical strategies, there is no ideal solution. From our point of view, any recommendation should therefore primarily focus on the strategies on how to expose the aneurysm as well as the inflow and outflow segments, how to handle the aneurysm, and how to avoid complications.

**Fig. 1:**
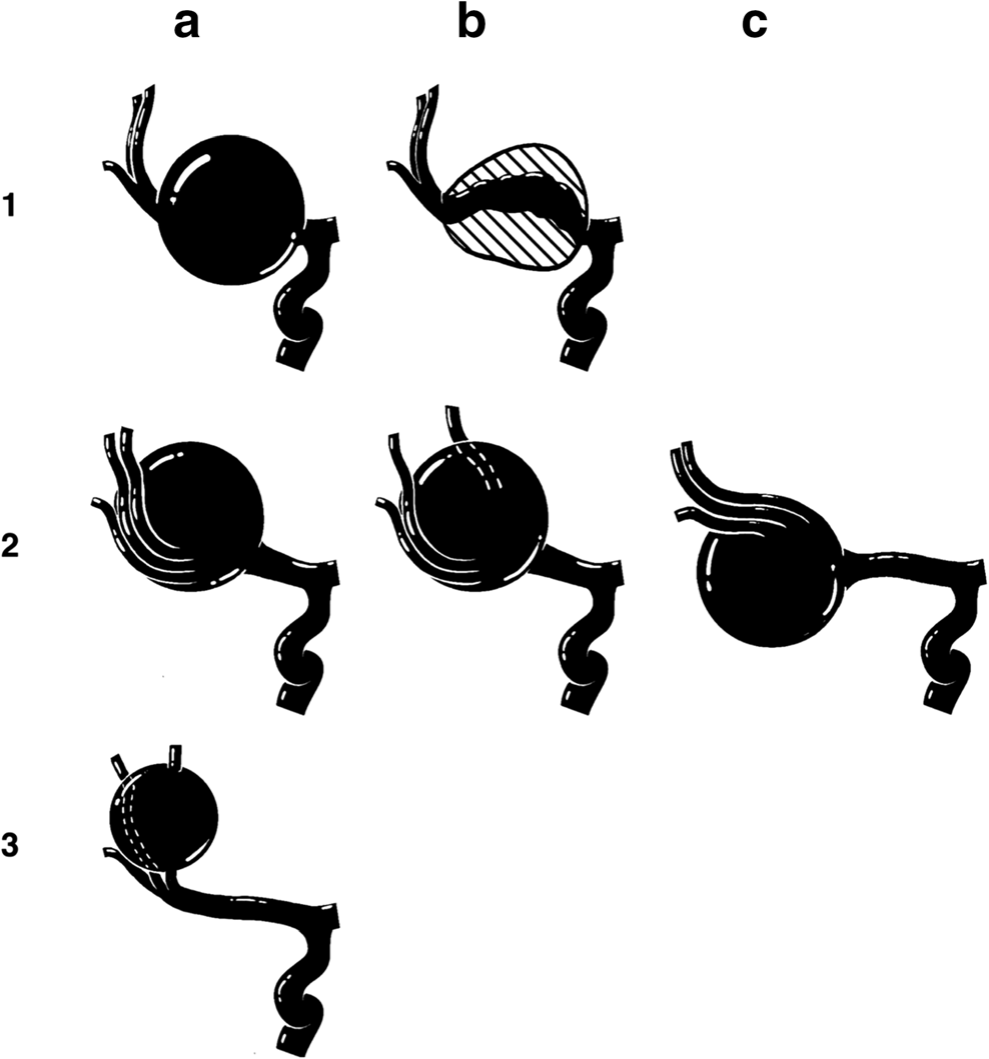
Overview of the classification for MCA aneurysm

Due to the lack of a classification addressing these aspects, we have proposed a classification for complex MCA aneurysms. We classified complex MCA aneurysms according to their localization into six categories: M1 aneurysms (type 1) subdivided into 1a without intra-aneurysmatic thrombosis and 1b with preexisting intra-aneurysmatic thrombosis, M1/M2 bifurcational aneurysms (type 2) subdivided into 2a with the M2 branches underneath the aneurysm in the ap view with good accessibility of the M2 branches, 2b where the aneurysm divides the M2 branches apart, and 2c where the M2 branches are hidden behind the aneurysm. Type 3 aneurysms are postbifurcational aneurysms without the involvement of the bifurcation (Fig. 1) [21]. Our series included 50 patients with complex aneurysms from our institution, in a retrospective monocentric design. To further validate our concept, this study now aimed at validating the Berlin classification for complex MCA aneurysms using an independent data set of patients with giant intracranial aneurysms of the MCA and determine its diagnostic reliability and interobserver applicability.

## Methods

We retrospectively reviewed 51 cases with giant MCA aneurysms from 18 centers participating in the international giant aneurysm registry [4]. We classified the aneurysms based on the ap view of the pretreatment DSA as previously reported. For testing the interrater reliability, four independent observers classified the 51 aneurysms according to our previously reported classification [21]. All observers were blinded for the clinical course of the patients and possible treatment. The observers had different levels of experience: rater 3 and 4 are vascular experienced neurosurgeons, and rater 1 and 2 are neurosurgical residents with vascular interest. We analyzed interrater reliability using the Fleiss’s Kappa; agreement was graded according to the Landis-Koch criteria (< 0 poor agreement, 0.0–0.20 slight agreement, 0.21–0.40 fair agreement, 0.41–0.60 moderate agreement, 0.61–0.80 substantial agreement, 0.81–1.0 almost perfect agreement) [15].

## Results

**Fig. 2:**
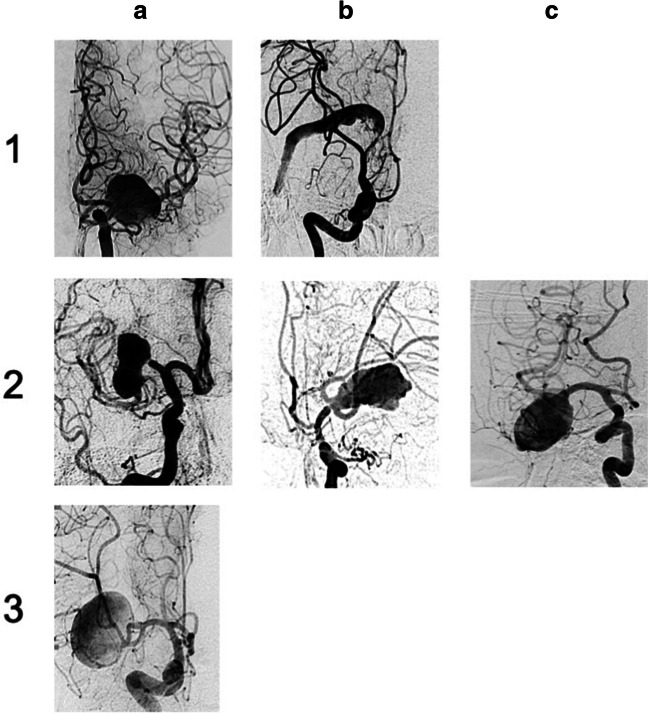
Examples for each type of aneurysm out of the giant aneurysm registry set of cases

The data set provided by the members of the international giant aneurysm registry consisted of 51 patients with giant aneurysms of the MCA. We were able to classify all aneurysms according to our preexisting classification system. Examples of the different types of aneurysms are given in Fig. 2.

**Fig. 3:**
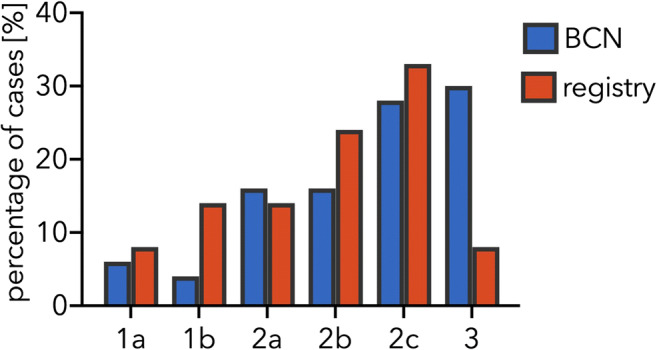
Percentage of cases for each type of aneurysm compared between the previously reported series of the Neurosurgical Department of the Charité University Medicine Berlin (BCN) compared with the series of cases provided by the giant aneurysm registry

Four (8%) aneurysms were classified as type 1a, 7 (14%) as type 1b, 7 (14%) as type 2a, 12 (24%) as type 2b, 17 (33%) as type 2c, and 4 (8%) as type 3. As Fig. 3 shows, the results differ from our previously published institutional series (1a: 6%, 1b: 4%, 2a: 16%, 2b: 16%, 2c: 28%, 3: 30%).

**Fig. 4:**
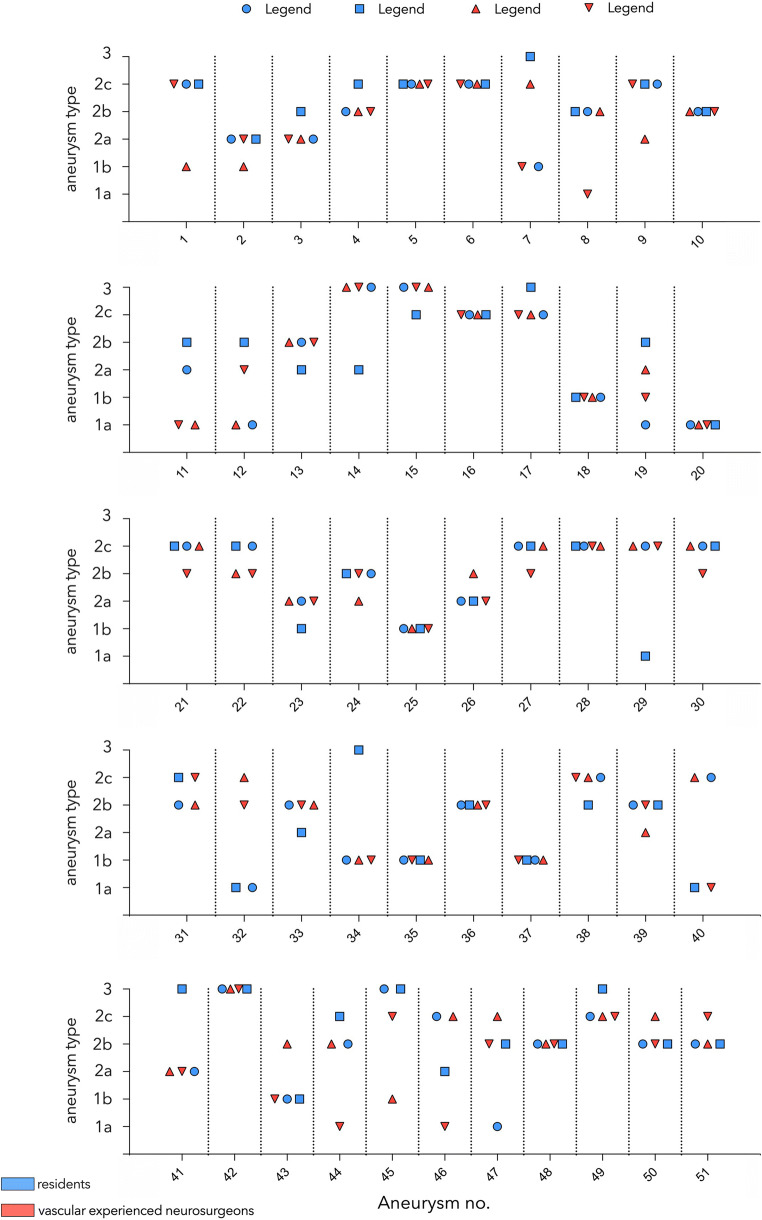
Rating of every aneurysm by the different observers

The interrater reliability was moderate with 0.419 (95% CI 0.418–0.42), and the different rater only agreed in 9 cases (Fig. 4).

None of the independent observers reported any difficulties applying the classification to the image set.

## Discussion

In this study, we demonstrate that the algorithmic structure can be applied to a separate series of 51 giant MCA aneurysms. The interrater reliability of 0.49 was moderate, and there was a large disagreement by grading the different aneurysm which might be due to the complexity of the aneurysms, since there does not seem to be an association with the level of experience. Since the observer in this study was restricted to AP and lateral view of the DSA, the disagreement can also be due to the need for detailed views and 3D reconstructions to evaluate such complex lesions. The distribution of the different types of MCA aneurysms differed from our previously reported series. One of the main reasons for this could be that in the previously reported series, not all aneurysms (56%) were giant, since we included all patients in whom an MCA aneurysm is treated with revascularization. Taking the MCA branching anatomy into account, type 3 aneurysms are limited in size, and the relatively low flow [3, 22] in the more distal MCA might not support the aneurysm growth to the same extent as in the more proximal segments. In general, the larger the size of the aneurysm, the greater is the chance that the bifurcation is involved, which is in line with previous reports on the distribution of giant aneurysms affecting the MCA [16].

The rupture rate of aneurysms increases with size [9, 12]. For untreated giant intracranial aneurysms, the 1-year rupture rate is 25.3% in non-cavernous intracranial aneurysms with a case fatality of 100% [5]. This underlines the need for treatment of these lesions. Our classification aims at improving the preoperative planning with an algorithmic approach to anticipate the relationship between the aneurysm, the M2 branches, and the incorporated perforators. Further, our classification focuses on predicting the accessibility of the inflow and outflow segments. Our classification system complements other reports focusing on the bypass technique at the pre-bifurcation, bifurcation, and postbifurcation levels [20].

In our classification system, M1 aneurysms are divided into aneurysms with (type 1b) and without (1a) intra-aneurysmatic thrombosis. The giant aneurysm cohort has more cases with intra-aneurysmatic thrombosis due to the large size. This categorization is important because in 1b aneurysms, the occlusion strategy can be more aggressive since perforators are likely already occluded, whereas in 1a aneurysms, complete trapping would cause infarction in the internal capsule. Since to the best of our knowledge the current literature does not provide information about the risk of complete trapping partial thrombosed M1 aneurysm, we recommend electrophysiological monitoring and test occlusion in all cases before trapping an MCA aneurysm. The categorization of the aneurysms affecting the bifurcation into type 2a-c helps estimating the chances of complete revascularization of the outflow segment, which the patient needs to be informed about and is essential for planning and successful trapping [21].

Although intraoperative techniques like indocyanine green videoangiography, flowmeter, or non-quantitative microdoppler allow assessment of blood flow in the recipient [1, 2, 7, 18], the decision of what kind of revascularization and aneurysm occlusion strategy should be used is not possible with these techniques. Further, it is of high importance to be able to perform the surgical planning beforehand, in order to counsel not only the patient but also the OR team about the planned surgical steps. Despite emerging 3D reconstruction techniques of CT angiography, and MR-based flow modeling, classical digital subtraction angiography still provides the most crucial information on deciding which treatment is best for complex aneurysms [8, 11, 17]. Nevertheless, 3D reconstruction techniques can help understanding the anatomy of the aneurysm.

Importantly, the structured classification that we propose serves as a simple, reliable, and cost-effective preoperative planning tool, which might help anticipate potential intraoperative pitfalls and facilitate the development of alternative surgical strategies before, instead of during the procedure.
